# Identifying inflammatory bowel disease patients at risk of metabolic dysfunction-associated fatty liver disease: usefulness of non-invasive steatosis predictive scores

**DOI:** 10.1186/s12876-023-02988-w

**Published:** 2023-12-13

**Authors:** Tiago Lima Capela, Vítor Macedo Silva, Marta Freitas, Cátia Arieira, Tiago Cúrdia Gonçalves, Francisca Dias de Castro, Joana Magalhães, José Cotter

**Affiliations:** 1 Hospital Senhora da Oliveira, Gastroenterology Department, Guimarães, Portugal; 2 Life and Health Sciences Research Institute (ICVS), School of Medicine, University of Minho, Braga/Guimarães, Braga, Portugal; 3 ICVS/3B’s, PT Government Associate Laboratory, Guimarães/Braga, Braga, Portugal

**Keywords:** Metabolic dysfunction-associated fatty liver disease, Inflammatory bowel disease, Nonalcoholic fatty liver disease, Non-invasive scores

## Abstract

**Background:**

Inflammatory bowel disease (IBD) patients have a higher risk of metabolic dysfunction-associated fatty liver disease (MAFLD) compared with the general population. However, it is not known whether available non-invasive hepatic steatosis scores are useful in predicting MAFLD in IBD patients. We aimed to analyze the performances of MAFLD screening score (MAFLD-S), Fatty Liver Index (FLI), Hepatic Steatosis Index (HSI) and Clinical Prediction Tool for NAFLD in Crohn’s Disease (CPN-CD), in identifying MAFLD in IBD patients.

**Methods:**

A cross-sectional study was carried out including consecutive adult IBD outpatients submitted to transient elastography (TE). MAFLD criteria were assessed, and hepatic steatosis (HS) was defined by a controlled attenuation parameter (CAP) >248 dB/m using TE. MAFLD-S, FLI, HSI, and CPN-CD were calculated and their accuracy for the prediction of MAFLD was evaluated through their areas under the receiver-operating characteristic (AUROC) curves.

**Results:**

Of 168 patients, body mass index ≥25, type 2 diabetes mellitus, dyslipidemia and arterial hypertension were present in 76 (45.2%), 10 (6.0%), 53 (31.5%), 20 (11.9%), respectively. HS was identified in 77 (45.8%) patients, of which 65 (84.4%) fulfilled MAFLD criteria. MAFLD-S (AUROC, 0.929 [95% CI, 0.888-0.971]) had outstanding and FLI (AUROC, 0.882 [95% CI, 0.830–0.934]), HSI (AUROC, 0.803 [95% CI, 0.736–0.871]), and CPN-CD (AUROC, 0.822 [95% CI, 0.753–0.890) had excellent discrimination in predicting MAFLD.

**Conclusions:**

MAFLD-S, FLI, HSI and CPN-CD scores can accurately identify MAFLD in IBD patients, allowing the selection of those in whom hepatic steatosis and metabolic risk factors assessment may be particularly beneficial.

## Introduction

Inflammatory bowel disease (IBD), comprising Crohn’s disease (CD) and ulcerative colitis (UC), is increasing across the globe with higher incidence and prevalence in North America and Western/Northern Europe [1].

Liver disease is a common comorbidity in IBD patients and nonalcoholic fatty liver disease (NAFLD) is an emerging cause of concern in this population [2]. NAFLD was recently redefined as metabolic dysfunction-associated fatty liver disease (MAFLD). Its diagnosis is based on histological, imaging or blood biomarkers evidence of hepatic steatosis (HS), in addition to one of the following three clinical criteria: overweight/ obesity, presence of type 2 diabetes mellitus (T2DM), or evidence of metabolic dysregulation, independently of the amount of alcohol consumed or other liver diseases [3]. Despite the novelty of this definition, its use has not been universally accepted worldwide, and most data on this condition come from studies using the previous definition [4].

In a recent meta-analysis, a pooled prevalence of 30.7% for NAFLD was found in patients with IBD worldwide, with the risk of NAFLD being two times higher in IBD patients compared with healthy subjects [5]. Additionally, no significant difference was observed in the odds ratio of NAFLD among CD patients compared with UC patients. The pooled prevalence of advanced liver fibrosis in IBD patients with NAFLD was 13.6%. Regardless of the influence of classic metabolic risk factors, IBD patients have an increased risk of NAFLD and liver fibrosis than general population [2].

The pathogenesis of MAFLD among the IBD population is not well understood [6]. Several studies have addressed the mechanisms underlying the association between MAFLD and IBD. While some of them indicate that the diagnosis of MAFLD in IBD patients is mainly due to the presence of well-established risk factors such as age, obesity, and T2DM, others have drawn attention to the role of IBD-related factors that may favor the development of MAFLD. These factors include the degree of inflammatory activity, the duration of the disease, history of IBD-related abdominal surgery and drug-mediated hepatotoxicity [7].

Nevertheless, IBD patients with concomitant MAFLD present a unique challenge. When compared to the general population, IBD patients experience higher mortality from NAFLD, with standardized mortality ratios of 2.26 and 2.82 in patients with UC and CD, respectively [8]. Additionally, NAFLD is associated with worse hospitalization outcomes in IBD patients, even after adjusting for metabolic factors [9]. NAFLD increases the risk of T2DM, cardiovascular diseases, and chronic kidney disease, not to mention the subsequent risk of developing liver cirrhosis and hepatocellular carcinoma [10].

Detecting HS is a mandatory criterion for MAFLD diagnosis, and ultrasound is the most widely used first-line diagnostic modality [3]. Nevertheless, has limited sensitivity for detection of mild (< 20%) steatosis, and its performance is suboptimal in individuals with body mass index (BMI) > 40 kg/m^2^ [11]. Measurement of controlled attenuation parameter (CAP) using vibration-controlled transient elastography (TE) is increasingly being undertaken in routine clinical practice for rapid and standardized HS detection. It has high applicability (> 95% of cases) and comparable accuracy as ultrasound for detecting HS, using biopsy as the reference standard [11].

Efforts have been made to develop a screening tool to identify HS and more specifically, NAFLD or MAFLD [12]. To avoid costs related to the mass implementation of imaging or TE studies, several non-invasive steatosis tests (NIT) have been developed. These include the fatty liver index (FLI), hepatic steatosis index (HSI), and, more recently, the Clinical Prediction Tool for NAFLD in Crohn’s Disease (CPN-CD), a score specifically developed for NAFLD prediction in CD patients, and the MAFLD screening score (MAFLD-S), developed for predicting MAFLD in the general population [13–16]. Although FLI and HSI have been independently validated in some populations, there is still scarce information regarding their use in MAFLD prediction and more specifically, their performance in predicting MAFLD in IBD patients [17]. In addition, CPN-CD and MAFLD-S lack external validation, and their applicability in IBD patients remains to be tested [15, 16].

Bearing in mind the clinical impact of the presence of MAFLD in IBD patients it is crucial to identify IBD patients at risk of MAFLD in whom HS and metabolic risk factors assessment may be particularly beneficial.

Recognizing this fact, we reasoned that a MAFLD screening program in IBD could be considered if patients are initially screened by a clinical non-invasive, readily available prediction tool, with a subset of at-risk patients undergoing ultrasound or TE to evaluate for HS and fibrosis and being evaluated for metabolic risk factors. Therefore, the objective of this study was to analyze the performances of MAFLD-S, FLI, HSI, and CPN-CD in predicting MAFLD in IBD patients.

## Methods

### Study design and patient selection

This study represents a secondary analysis of a cohort of IBD patients included in a previously published article [18]. Our group conducted a cross-sectional study in which consecutive IBD outpatients were submitted to TE in a University-Affiliated Hospital between January and March 2017. Eligible patients were older than 18 years with an IBD diagnosis (according to European and American guidelines) and with serum parameters obtained within 2 weeks of our observation [19, 20]. Patients with known liver disease, including alcoholic, autoimmune, viral, and clinical diagnosis of an alternative metabolic/toxic liver disease were excluded. Moreover, patients with heavy alcohol habits (> 20 g/day for women or > 30 g/day for men), TE measurement failure (no valid measurements after at least 10 attempts), unreliable TE measurements (interquartile range to median ratio (IQR/M) > 30%) or missing data in medical records were also excluded. Contrary to previous published article, we did not exclude patients exposed to glucocorticoids or immunomodulators (including azathioprine, 6-mercaptopurine or methotrexate), as recent meta-analysis findings did not identify them as significant risk factors for NAFLD in IBD patients [5]. The need for informed consent was waived by the Senhora da Oliveira Hospital’s Ethics Committee due to the retrospective nature of the study. Our report adheres to the Strengthening the Reporting of Observational Studies in Epidemiology (STROBE) guidelines [21].

### Data collection and variables definition

Several data were retrospectively collected from electronic medical records.

Demographic information (age, sex, race), body weight (in kilograms), height (in centimeters), BMI (in kg/m^2^), waist circumference (in centimeters, measured at a level midway between the lowest rib and the iliac crest) and tobacco use (current or previous) data were gathered.

Data regarding IBD type (CD or UC), family history of IBD, age at diagnosis, duration of disease (in months between diagnosis and TE), location and behavior of CD according to Montreal classification, presence of perianal disease, distribution of UC, current medication used, namely mesalazine (oral or topical), glucocorticoids (prednisone, prednisolone, budesonide), immunomodulators (azathioprine, 6-mercaptopurine, methotrexate), biological therapy (infliximab, adalimumab, vedolizumab, ustekinumab) and previous IBD-related abdominal surgery data were also collected [22].

Some comorbidities including the presence of overweight/obesity, arterial hypertension, impaired fasting glycemia, T2DM, and dyslipidemia were assessed and defined according to the criteria for MAFLD diagnosis [3].

All serum parameters, such as hemoglobin (g/dL), leucocytes (× 10^3^ per cubic millimeter), platelets (× 10^3^ per cubic millimeter), fasting plasma glucose (FPG) (mg/dL), aspartate aminotransferase (AST) (IU/L), alanine aminotransferase (ALT) (IU/L), γ-glutamyltransferase (G-GT) (IU/L), alkaline phosphatase (AP) (IU/L), albumin (g/dL), total and direct bilirubin (mg/dL), total cholesterol (TC) (mg/dL), low-density cholesterol (LDL) and high-density lipoprotein cholesterol (HDL) (mg/dL), triglycerides (mg/dL), erythrocyte sedimentation rate (ESR) (mm/hour) and C-reactive protein (CRP) (mg/L), were obtained after a 12-h overnight fasting, within 2 weeks of TE. Stool samples for calprotectin measurement (ug/g) were collected from the first bowel movement of the day.

**Fig. 1:**
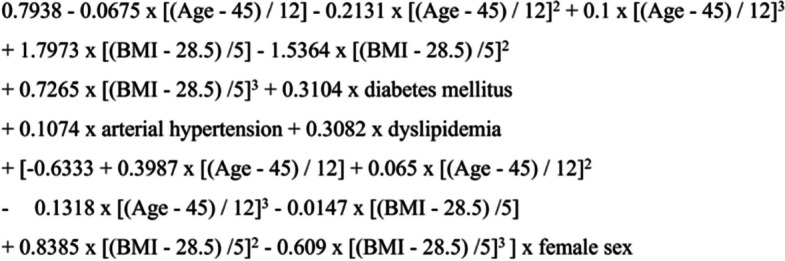
Original formula of metabolic dysfunction-associated fatty liver disease screening score [16]. BMI body mass index

**Fig. 2:**
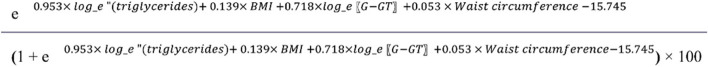
Original formula of fatty liver index [13]. BMI body mass index, G-GT γ-glutamyltransferase

**Fig. 3:**
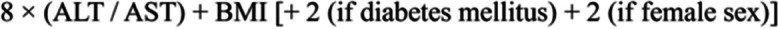
Original formula of hepatic steatosis index [14]. ALT alanine aminotransferase, AST aspartate aminotransferase, BMI body mass index

**Fig. 4:**
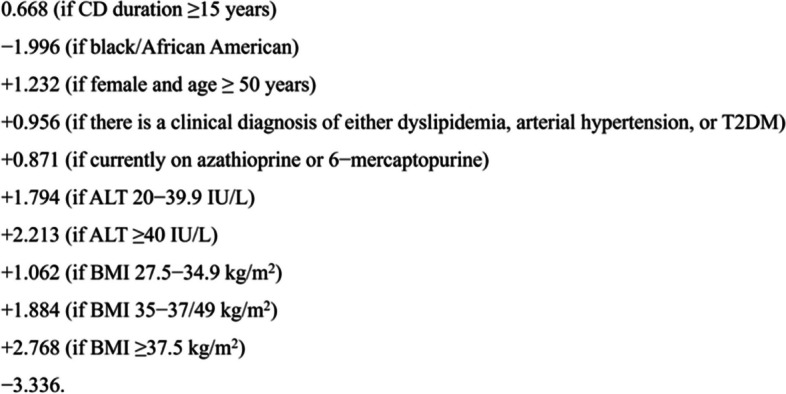
Original formula of Clinical Prediction Tool for NAFLD in Crohn’s Disease [15]. ALT alanine aminotransferase, AST aspartate aminotransferase, BMI body mass index, CD Crohn’s Disease, T2DM type 2 diabetes mellitus

MAFLD-S, FLI, HSI and CPN-CD were calculated according to the original formulas (Figs. 1, 2, 3 and 4) [13–16].

TE (FibroScan; Echosens, Paris, France) was performed on patients following a minimum 2-h fasting, as recommended by manufacturer [23]. Quantification of liver stiffness measurement (LSM, in kilopascal (kPa)), controlled attenuation parameter (CAP, in decibels per meter (dB/m)) and IQR/M were performed using the M probe (or XL probe when unreliable TE measures were obtained with M probe), on the right lobe of the liver through 9–11th intercostal space on the middle axillary line, with the patient lying in a dorsal position and the right arm in maximal abduction. To be considered a valid and reliable examination, ten or more successful acquisitions were performed, and IQR/M had to be less than 30% [24]. The operator was experienced, had undergone formal training, and performed at least 500 examinations before this study.

MAFLD diagnosis was assessed according to the original criteria: evidence of HS in addition to one of the following three criteria, namely overweight/ obesity, presence of T2DM, or evidence of metabolic dysregulation including at least two of the described metabolic risk abnormalities [3]. Patients fulfilling MAFLD criteria without the presence overweight/ obesity were considered as Lean-MAFLD [25]. We defined HS as a CAP > 248 dB/m [26]. Overweight/obesity was defined as BMI ≥ 25 kg/m^2^ in Caucasians or BMI ≥ 23 kg/m^2^ in Asians, T2DM as FPG ≥ 126 mg/dL or specific drug treatment. The following metabolic risk abnormalities were also considered: waist circumference ≥ 102/88 cm in Caucasian men and women (or ≥ 90/80 cm in Asian men and women), blood pressure ≥ 130/85 mmHg or specific drug treatment, plasma triglycerides ≥ 150 mg/dl or specific drug treatment, plasma HDL < 40 mg/dL for men and < 50 mg/dL for women or specific drug treatment, impaired fasting glycemia (FPG of 100–125 mg/dL) and plasma CRP > 2 mg/L [3].

### Statistical analysis

The Statistical Package for Social Sciences program version 26 (IBM Corporation, Armonk, NY) was used for analysis. Categorical variables were described using absolute frequencies and percentages. If necessary, chi-square test or Fisher’s exact test was used to compare categorical variables. Depending on the normality tests, continuous variables were expressed as mean ± standard deviation (SD) or median (interquartile range (IQR)). Means were compared between distinct groups using independent samples t-test. When applicable, non-parametric tests were performed. We assessed the performance of MAFLD-S, FLI, HSI and CPN-CD in predicting MAFLD in IBD patients both in general and separately by IBD type (CD and UC). The discriminatory ability of the scores was evaluated using receiver operator characteristic (ROC) curve analysis, with determination of the area under the curve (AUROC) and its corresponding 95% confidence interval (CI). According to the Hosmer and Lemeshow guidelines for predictive ability evaluation, an AUROC of 0.5000–0.699 indicated a poor ability, 0.7000–0.799 indicated an acceptable ability, 0.800–0.899 indicated an excellent ability, and 0.900–0.999 indicated an outstanding ability [27]. In addition, sensitivity (Se), specificity (Sp), positive predictive value (PPV) and negative predictive value (NPV) of each score were calculated, as well as the respective Youden index. The most suitable cut-off value for each score was determined as having the highest Youden index [28]. Statistical significance was defined as *P* < 0.05.

## Results

**Fig. 5:**
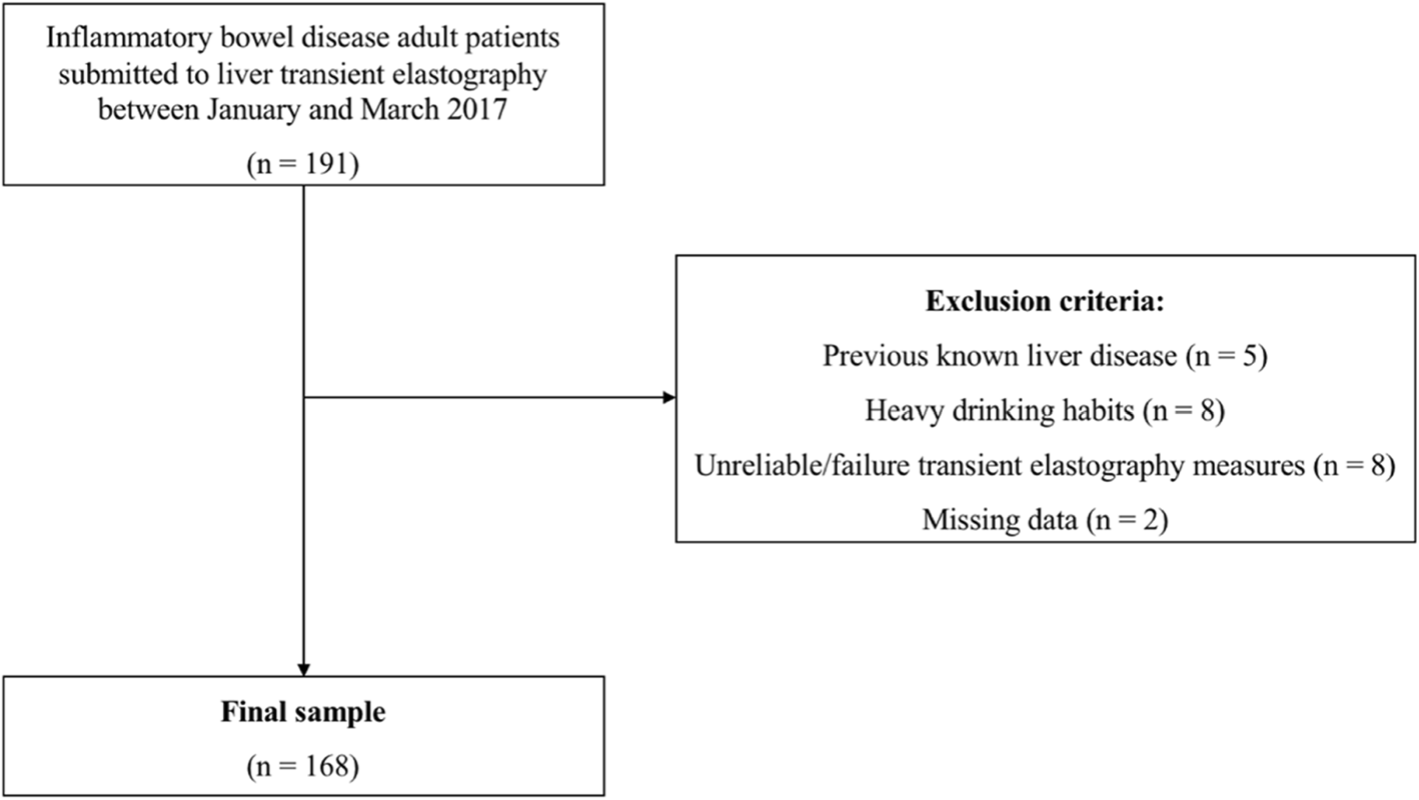
Flowchart displaying the selection of patients in the study cohort

After applying exclusion criteria, our final sample included 168 patients with IBD (Fig. 5).

The main demographic, clinical, laboratorial and TE findings are presented in Tables 1 and 2. Among the 168 patients, all of whom were white, 90 (53.6%) were female, with a mean age of 40.1 ± 12.6 years.

**Table 1:** Demographic, clinical, laboratory and transient elastography findings according to the inflammatory bowel disease type (Crohn’s disease and ulcerative colitis)

**Variables**	**All patients** (*n* = 168)	**Crohn’s disease** (*n* = 107, 63.7%)	**Ulcerative colitis** (*n* = 61, 36.3%)	***P* value**
**Demographics**				
Mean **age ± **SD – years	40.1 ± 12.6	38.4 ± 11.9	43.3 ± 13.4	0.234
**White race**– n, (%)	168 (100)	107 (100)	61 (100)	1.0
**Female sex** – n, (%)	90 (53.6)	57 (53.3)	33 (54.1)	1.0
**IBD related factors**				
**Montreal classification** – n, (%)				
A1, A2, A3	-	8(7.5); 75(70.1); 24(22.4)	-	-
L1, L2, L3	-	56 (52.3); 10(9.3); 41(38.3)	-	-
L4	-	0	-	-
B1, B2, B3	-	48(44.9);36(33.6);23(21.5)	-	-
Perianal disease	-	32 (29.9)	0	** < 0.001**
**Disease extension** – n, (%)				
E1				
E2	-	-	17 (27.9)	
E3	-	-	18 (29.5)	-
**Mean Age at IBD diagnosis** ±	-	-	26 (42.6)	-
SD– years	33.3 ± 12.3	30.9 ± 11.6	37.3 ± 12.6	-
**Median IBD duration** (IQR) –	72.0 (84.0)	72.0 (108)	72.0 (78)	0.454
months	56 (33.3)	38 (35.5)	18 (29.5)	0.851
**Tobacco use** – n, (%)	34 (20.2)	34 (31.8)	0	0.497
**IBD-related abdominal surgery** – n, (%)	16 (9.5)	10 (9.3)	6 (9.8)	** < 0.001**
**Family history of IBD** – n, (%)	74 (44.0)	22 (20.6)	52 (85.2)	1.000
**Mesalazine** – n, (%)	82 (48.8)	60 (56.1)	22 (36.1)	** < 0.001**
Immunomodulators* – n, (%)	6 (3.6)	3 (2.8)	3 (4.9)	**0.024**
**Glucocorticoids** – n, (%)	68 (40.5)	49 (45.8)	19 (31.1)	1.000
**Biologic therapy** – n, (%)				0.073
**Anthropometric data**				
Mean **weight** ± SD – kg	69.1 ± 12.5	70.7 ± 13.5	66.4 ± 10.2	**0.033**
Mean **height** ± SD – cm	166.1 ± 9.2	167.0 ± 9.5	164.6 ± 8.5	0.106
Mean **BMI** ± SD – kg/m2	25.1 ± 4.0	25.5 ± 4.2	24.5 ± 3.4	0.122
Mean **waist circumference** ± SD – cm	87.5 ± 11.5	88.4 ± 12.1	85.9 ± 10.1	0.183
**Laboratorial data**				
Mean **Hemoglobin** ± SD – g/dL	14.0 ± 1.3	13.9 ± 1.1	14.1 ± 1.5	0.299
Median **Leucocyte count** (IQR) – x10^3^μL	6.9 (2.7)	6.8 (2.6)	7.2 (3.0)	0.872
Mean **Platelet count** ± SD – x10^3^μL	270.7 ± 80.9	272.6 ± 91.0	267.2 ± 84.7	0.678
Median **FPG** (IQR) – mg/dL	89.0 (16.8)	88.0 (16.0)	89.0 (15.5)	0.626
Median **AST** (IQR) – IU/L	18.0 (10.0)	18.0 (10.0)	16.0 (12.0)	0.231
Median **ALT** (IQR) – IU/L	26.0 (16.0)	26.0 (16.0)	26.0 (17.5)	0.9
Median **G-GT** (IQR) – IU/L	22.0 (19.0)	22.0 (21.0)	24.0 (18.5)	0.575
Median **AP** (IQR) – IU/L	64.0 (20.8)	64.0 (21.0)	64.0 (22.0)	0.449
Median **albumin** (IQR) – g/dL	3.8 (0.5)	3.8 (0.5)	4.0 (0.5)	0.052
Median **total bilirubin** (IQR) – mg/dL	0.5 (0.3)	0.5 (0.4)	0.5 (0.3)	0.665
Median **direct bilirubin** (IQR) – mg/dL	0.1 (0.1)	0.1 (0.1)	0.1 (0.1)	0.537
Mean **total cholesterol** ± SD – mg/dL	175.5 ± 13.1	175.2 ± 35.2	175.9 ± 34.6	0.905
Mean **LDL** ± SD – mg/dL	97.0 ± 28.0	94.0 ± 28.0	102.3 ± 27.7	0.066
Mean **HDL** ± SD – mg/dL	54.3 ± 13.6	54.9 ± 14.2	53.2 ± 12.5	0.459
Median **triglycerides** (IQR) – mg/dL	109.0 (70.8)	117.0 (72.0)	92.0 (58.0)	**0.026**
Median **CRP** (IQR) – mg/L	2.9 (2.0)	2.9 (2.8)	2.9 (0.3)	0.066
Median **ESR** (IQR) – mm	10.0 (12.8)	10.0 (13.0)	9.0 (12.5)	0.578
Median **Calprotectin** (IQR) – μg/g	184.0 (559.3)	184.0 (404.0)	197 (741.5)	0.716
**Steatosis scores**				
Median **MAFLD-S** (IQR)	-1.9 (4.1)	-1.6 (4.2)	-2.1 (4.2)	0.245
Mean **FLI** ± SD	33.9 ± 26.3	36.6 ± 27.3	29.2 ± 23.9	0.079
Median **HSI** (IQR)	38.1 (8.7)	38.0 (9.0)	39.4 (9.1)	0.338
Mean **CPN-CD** ± SD	- 0.6 ± 1.8	- 0.5 ± 1.5	- 0.7 ± 1.3	0.314
**TE**				
Median **LSM** (IQR) – kPa	4.6 (1.8)	4.5 (1.7)	4.7 (1.9)	0.567
Mean **IQR/M** ± SD – %	14.0 ± 8.8	14.2 ± 6.0	14.5 ± 6.3	0.213
Mean **CAP** ± SD – dB/m	249.0 (55.9)	249.8 ± 59.1	247.6 ± 50.3	0.807
**MAFLD criteria**				
**MAFLD** – n, (%)	65 (38.7)	46 (43.0)	19 (31.1)	0.141
**Lean MAFLD** – n, (%)	6 (3.6)	3 (2.8)	3 (4.9)	0.669
**CAP > 248 dB/m** – n, (%)	77 (45.8)	52 (48.6)	25 (41.0)	0.421
**Overweight/Obesity** – n, (%)	76 (45.2)	55 (51.4)	21 (34.4)	**0.037**
**T2DM** – n, (%)	10 (6.0)	5 (4.7)	5 (8.2)	0.499
**Impaired fasting glycemia** – n, (%)	21 (12.5)	12 (11.2)	9 (14.8)	0.628
**Abnormal waist circumference** – n, (%)	51 (30.4)	38 (35.5)	13 (21.3)	0.058
**Dyslipidemia** – n, (%)	53 (31.5)	29 (27.1)	24 (39.3)	0.121
**Arterial Hypertension** – n, (%)	20 (11.9)	10 (9.3)	10 (16.4)	0.217
**Plasma CRP > 2 mg/L** – n, (%)	58 (34.5)	42 (39.3)	16 (26.2)	0.095

*ALT* Alanine aminotransferase, *AP* Alkaline phosphatase, *AST* Aspartate aminotransferase, *BMI* Body mass index, *CAP* Controlled attenuated parameter, *CPN-CD* Clinical Prediction Tool for NAFLD in Crohn’s Disease, *CRP* C-reactive protein, *ESR* Erythrocyte sedimentation rate, *FLI* Fatty liver index, *FPG* Fasting plasma glucose, *G-GT* γ-glutamyltransferase, *HDL* High-density lipoprotein cholesterol, *HSI* Hepatic steatosis index, *IBD* Inflammatory bowel disease, *IQR/M* Interquartile range to median ratio, *LDL* Low-density cholesterol, *LSM* Liver stiffness measure, *MAFLD* Metabolic associated fatty liver disease, *MAFLD-S* Metabolic associated fatty liver disease screening score, *T2DM* Type 2 diabetes mellitus,

**Table 2:** Demographic, clinical, laboratory and transient elastography findings according to the presence of metabolic associated fatty liver disease

**Variables**	**All patients **(*n* = 168)	**MAFLD **(*n* = 65, 38.7%)	**Non-MAFLD** (*n* = 103, 61.3%)	***P* value**
**Demographics**				
Mean **age** ± SD – years	40.1 ± 12.6	46.3 ± 12.3	36.3 ± 11.2	** < 0.001**
**White race**– n, (%)	168 (100)	65(100)	103(100)	1.0
**Female sex** – n, (%)	90 (53.6)	28 (43.1)	62 (60.2)	**0.039**
**IBD-related factors**				
**Crohn’s disease** – n, (%)		46 (70.8)	61 (59.2)	0.141
**Montreal classification** – n, (%)				
A1	8(7.5)	2 (4.3)	6 (9.8)	0.462
A2	75(70.1)	29 (63.0)	46 (75.4)	0.203
A3	24(22.4)	15 (32.6)	9 (14.8)	**0.036**
L1	56 (52.3)	26 (56.6)	30 (49.2)	0.558
L2	10(9.3)	3 (6.5)	7 (11.5)	0.510
L3	41(38.3)	17 (37.0)	24 (39.3)	0.843
L4	0	0	0	-
B1	48(44.9)	20 (43.5)	28 (45.9)	0.846
B2	36(33.6)	15 (32.6)	21 (34.4)	1.000
B3	23(21.5)	11 (23.9)	12 (19.7)	0.640
Perianal disease	33.3 (12.3)	16 (24.6)	16 (15.5)	0.161
**Disease extension** – n, (%)				
E1	17 (27.9)	4 (21.1)	13 (31.0)	0.544
E2	18 (29.5)	7 (36.8)	11 (26.2)	0.545
E3	26 (42.6)	8 (42.1)	18 (42.9)	1.000
**Mean Age at IBD diagnosis** ± SD – years	33.3 ± 12.3	38.6 ± 12.6	29.9 ± 10.9	** < 0.001**
**Median IBD duration** (IQR) – months	72.0 (84.0)	84 (89)	72.0 (96)	0.207
**Tobacco use** – n, (%)	56 (33.3)	22 (33.8)	34 (33.0)	1.000
**IBD-related abdominal surgery** – n, (%)	34 (20.2)	19 (29.2)	15 (14.6)	**0.029**
**Family history of IBD** – n, (%)	16 (9.5)	4 (6.2)	12 (11.7)	0.289
**Mesalazine** – n, (%)	74 (44.0)	27 (41.5)	47 (45.6)	0.635
**Immunomodulators** – n, (%)	82 (48.8)	36 (55.4)	46 (44.6)	0.156
**Glucocorticoids** – n, (%)	6 (3.6)	3 (4.6)	3 (2.9)	0.678
**Biologic therapy** – n, (%)	68 (40.5)	22 (33.8)	46 (44.7)	0.197
**Anthropometric data**				
Mean **weight** ± SD – kg	69.1 ± 12.5	78.4 ± 10.0	63.3 ± 10.3	** < 0.001**
Mean **height** ± SD – cm	166.1 ± 9.2	165.7 ± 9.2	166.4 ± 9.2	0.621
Mean **BMI** (SD) – kg/m2	25.1 ± 4.0	28.6 ± 3.3	22.9 ± 2.6	** < 0.001**
Mean **waist circumference** (SD) – cm	87.5 ± 11.5	96.4 ± 9.7	81.8 ± 8.6	** < 0.001**
**Laboratorial data**				
Mean **Hemoglobin** ± SD – g/dL	14.0 ± 1.3	14.1 ± 1.3	13.9 ± 1.3	0.194
Median **Leucocyte count** (IQR) – x10^3^μL	6.9 (2.7)	6.6 (2.6)	7.2 (2.7)	0.384
Mean **Platelet count** ± SD – x10^3^μL	270.7 ± 80.9	264.0 ± 79.3	274.9 ± 82.1	0.396
Median **FPG** (IQR) – mg/dL	89.0 (16.8)	95.0 (18.0)	87.0 (16.0)	** < 0.001**
Median **AST** (IQR) – IU/L	18.0 (10.0)	19.0 (9.5)	17.0 (10.0)	0.103
Median **ALT** (IQR) – IU/L	26.0 (16.0)	29.0 (16.5)	23.0 (15.0)	**0.001**
Median **G-GT** (IQR) – IU/L	22.0 (19.0)	28.0 (21.5)	19.0 (18.0)	** < 0.001**
Median **AP** (IQR) – IU/L	64.0 (20.8)	64.0 (20.5)	64.0 (22.0)	0.713
Median **albumin** (IQR) – g/dL	3.8 (0.5)	3.9 (0.4)	3.9 (0.6)	0.499
Median **total bilirubin** (IQR) – mg/dL	0.5 (0.3)	0.5 (0.4)	0.5 (0.4)	0.717
Median **direct bilirubin** (IQR) – mg/dL	0.1 (0.1)	0.1 (0.1)	0.1 (0.1)	0.896
Mean **total cholesterol** ± SD – mg/dL	175.5 ± 13.1	181.5 ± 35.0	171.7 ± 34.4	0.076
Mean **LDL** ± SD – mg/dL	97.0 ± 28.0	102.0 ± 28.8	93.9 ± 27.3	0.068
Mean **HDL** ± SD – mg/dL	54.3 ± 13.6	52.2 ± 12.9	55.5 ± 13.9	0.123
Median **triglycerides** (IQR) – mg/dL	109.0 (70.8)	120.0 (62.5)	96.0 (66.0)	**0.006**
Median **CRP** (IQR) – mg/L	2.9 (2.0)	2.9 (1.5)	2.9 (2.8)	0.760
Median **ESR** (IQR) – mm	10.0 (12.8)	10.0 (12.5)	10.0 (12.0)	0.477
Median **Calprotectin** (IQR) – μg/g	184.0 (559.3)	172 (444.5)	202 (664)	0.264
**Steatosis scores**				
Median **MAFLD-S** (IQR)	-1.9 (4.1)	0.2 (1.8)	-3.6 (3.8)	** < 0.001**
Mean **FLI** ± SD	33.9 ± 26.3	55.3 ± 23.6	20.4 ± 17.5	** < 0.001**
Median **HSI** (IQR)	38.1 (8.7)	42.4 (8.8)	36.1 (6.44)	** < 0.001**
Mean **CPN-CD** ± SD	- 0.6 ± 1.8	0.3 ± 1.4	-1.2 ± 1.1	** < 0.001**
**TE**				
Median **LSM** (IQR) – kPa	4.6 (1.8)	4.8 (2.1)	4.5 (1.9)	0.078
Mean **IQR/M** ± SD – %	14.0 ± 8.8	14.0 ± 6.0	15.1 ± 6.1	0.247
Mean **CAP** ± SD – dB/m	249.0 ± 55.9	300.6 ± 36.0	216.4 ± 39.2	** < 0.001**
**MAFLD criteria**				
**CAP > 248 dB/m** – n, (%)	77 (45.8)	65 (100)	12 (11.7)	** < 0.001**
**Overweight/Obesity** – n, (%)	76 (45.2)	59 (90.8)	17 (16.5)	** < 0.001**
**T2DM** – n, (%)	10 (6.0)	8 (12.3)	2 (1.9)	**0.014**
**Impaired fasting glycemia** – n, (%)	21 (12.5)	10 (15.4)	11 (10.7)	0.473
**Abnormal waist circumference** – n, (%)	51 (30.4)	32 (49.2)	19 (18.4)	** < 0.001**
**Dyslipidemia** – n, (%)	53 (31.5)	30 (46.2)	23 (22.3)	**0.002**
**Arterial Hypertension** – n, (%)	20 (11.9)	16 (24.6)	4 (3.9)	** < 0.001**
**Plasma CRP > 2 mg/L** – n, (%)	58 (34.5)	23 (35.4)	35 (34.0)	0.869

*ALT* Alanine aminotransferase, *AP* Alkaline phosphatase, *AST* Aspartate aminotransferase, *BMI* Body mass index, *CAP* Controlled attenuated parameter, *CPN-CD* Clinical Prediction Tool for NAFLD in Crohn’s Disease, *CRP* C-reactive protein, *ESR* Erythrocyte sedimentation rate, *FLI* Fatty liver index, *FPG* Fasting plasma glucose, *G-GT* γ-glutamyltransferase, *HDL* High-density lipoprotein cholesterol, *HSI* Hepatic steatosis index, *IBD* Inflammatory bowel disease, *IQR/M* Interquartile range to median ratio, *LDL* Low-density cholesterol, *LSM* Liver stiffness measure, *MAFLD* Metabolic associated fatty liver disease, *MAFLD-S* Metabolic associated fatty liver disease screening score, *T2DM* Type 2 diabetes mellitus

The majority of patients had CD (*n* = 107, 63.7%), more specifically, A2 (*n* = 75, 70.1%), L1 (*n* = 56, 52.3%), B1 (*n* = 48, 44.9%), according to Montreal classification. Regarding UC patients, most had pancolitis (*n* = 26, 42.6%). Compared to UC, CD patients had more frequently perianal disease (29.9% vs 0%, *P* < 0.001) and history of IBD-related abdominal surgery (31.8% vs 0%, *P* < 0.001), and were taking immunomodulators more frequently (56.1% vs 36.1%, *P* = 0.024). In contrast, CD patients were taking mesalazine less frequently than UC patients (20.6% vs 85.2%, *P* < 0.001). Furthermore, CD patients had a higher body weight (70.7 ± 13.5 vs 66.4 ± 10.2, *P* = 0.033) and had higher levels of plasma triglycerides (117.0 (72.0) vs 92.0 (58.0), *P* = 0.026) compared to UC patients.

Regarding NIT, a median MAFLD-S of -1.9 (4.1), a mean FLI of 33.9 ± 26.3, a median HSI of 38.1 (8.7) and a mean CPN-CD of—0.6 ± 1.8 was obtained for all patients independently of IBD type. MAFLD-S [-1.6 (4.2) vs -2.1 (4.2), *P* = 0.245], FLI (36.6 ± 27.3 vs 29.2 ± 23.9, *P* = 0.079), HSI [38.0 (9.0) vs 39.4 (9.1), *P* = 0.338] and CPN-CD (- 0.5 ± 1.5 vs—0.7 ± 1.3, *P* = 0.314) were not significantly different between CD and UC patients.

In general, IBD patients had a mean CAP of 249.0 ± 55.9 dB/m, median LSM of 4.6 (1.8) kPa and mean IQR/M of 14.0 ± 8.8%. Neither CAP (249.8 ± 59.1 vs 247.6 ± 50.3, *P* = 0.807), LSM [4.5 (1.7) vs 4.7 (1.9), *P* = 0.567] nor IQR/M (14.2 ± 6.0 vs 14.5 ± 6.3, *P* = 0.213) were significantly different between CD and UC patients.

The overall prevalence of HS, BMI ≥ 25 kg/m^2^, T2DM, impaired fasting glycemia, abnormal waist circumference, dyslipidemia and arterial hypertension was 45.8%, 45.2%, 6.0%, 12.5%, 30.4%, 31.5%, 11.9%, respectively. Among these variables, only prevalence of BMI ≥ 25 kg/m^2^ was significantly different between CD and UC patients (51.4% vs 34.4%, *P* = 0.037).

MAFLD criteria were fulfilled in 65 (38.7%) patients, with 6 (9.2%) meeting the criteria for lean-MAFLD. The prevalence of MAFLD was not significantly different between CD and UC patients (43.0% vs 31.1%, *P* = 0.141). Compared to non-MAFLD, MAFLD patients were older (46.3 ± 12.3 vs 36.3 ± 11.2, *P* < 0.001) and more frequently male (56.9% vs 39.8%, *P* = 0.039). Additionally, they were older at the time of IBD diagnosis (38.6 ± 12.6 vs 29.9 ± 10.9, *P* < 0.001), more likely to have a history of IBD-related abdominal surgery (29.2% vs 14.6%, *P* = 0.029), and exhibited higher G-GT [28.0 (21.5) vs 19.0 (18.0), *P* < 0.001], FPG [95.0 (18.0) vs 87.0 (16.0), *P* < 0.001], and triglycerides levels [120.0 (62.5) vs 96.0 (66.0), *P* = 0.006].

**Fig. 6:**
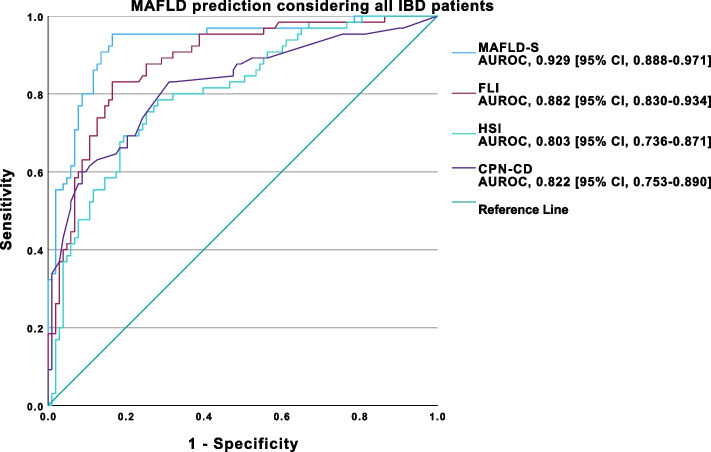
ROC curve analysis of MAFLD-S, FLI, HSI and CPN-CD performance for MAFLD prediction in all IBD patients

MAFLD-S (AUROC, 0.929 [95% CI, 0.888–0.971]) demonstrated outstanding performance, while FLI (AUROC, 0.882 [95% CI, 0.830–0.934]), HSI (AUROC, 0.803 [95% CI, 0.736–0.871]), and CPN-CD (AUROC, 0.822 [95% CI, 0.753–0.890]) exhibited excellent discriminative ability in predicting MAFLD (Fig. 6.). The optimal cut-off obtained for MAFLD-S was -1.6 (Se 95.4%, Sp 83.5%, NPV 96.6%, PPV 78.5%), 32.87 (Se 83.1%, Sp 83.5%, NPV 88.7%, PPV 76.1%) for FLI, 38.64 (Se 78.5%, Sp 71.8%, NPV 84.1%, PPV 63.7%) for HSI, and -0.58 (Se 83.1%, Sp 68.9%, NPV 86.9%, PPV 62.9%) for CPN-CD.

**Fig. 7:**
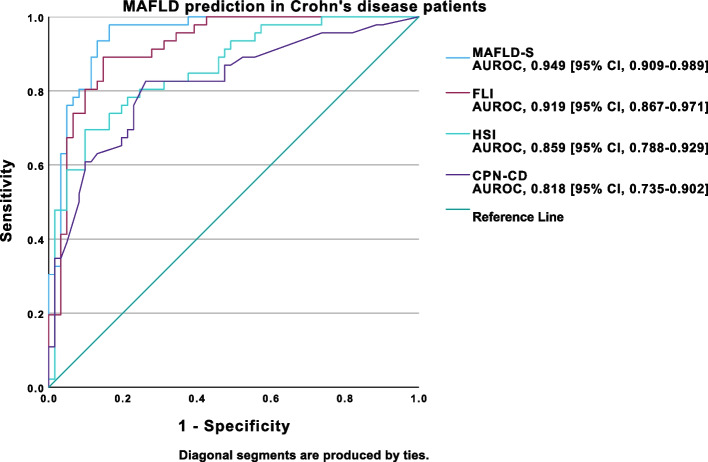
ROC curve analysis of MAFLD-S, FLI, HSI and CPN-CD performance for MAFLD prediction in Crohn’s disease patients

In CD patients, MAFLD-S (AUROC, 0.949 [95% CI, 0.909–0.989]) and FLI (AUROC, 0.919 [95% CI, 0.867–0.971]) demonstrated outstanding performance, while HSI (AUROC, 0.859 [95% CI, 0.788–0.929]) and CPN-CD (AUROC, 0.818 [95% CI, 0.735–0.902]) exhibited excellent discriminative ability in predicting MAFLD (Fig. 7.). In these patients, the best cut-off obtained for MAFLD-S was -1.6 (Se 97.8%, Sp 83.6%, NPV 98.2%, PPV 80.4%), 32.59 (Se 89.1%, Sp 85.2%, NPV 91.2%, PPV 82.0%) for FLI, 40.2 (Se 69.6%, Sp 90.2%, NPV 79.7%, PPV 84.3%) for HSI and -0.58 (Se 82.6%, Sp 73.8%, NPV 84.9%, PPV 70.4%) for CPN-CD.

**Fig. 8:**
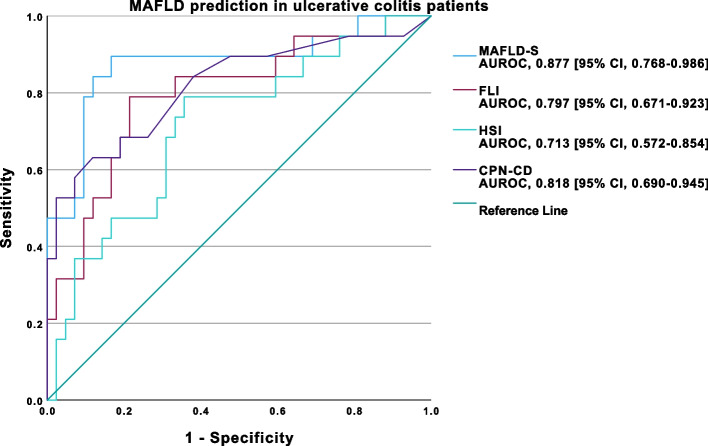
ROC curve analysis of MAFLD-S, FLI, HSI and CPN-CD performance for MAFLD prediction in ulcerative colitis patients

In UC patients, MAFLD-S (AUROC, 0.877 [95% CI, 0.768–0.986]) and CPN-CD (AUROC, 0.818 [95% CI, 0.690–0.945]) demonstrated excellent performance, while FLI (AUROC, 0.797 [95% CI, 0.671–0.923]) and HSI (AUROC, 0.713 [95% CI, 0.572–0.854]) exhibited acceptable discriminative ability in predicting MAFLD (Fig. 8.). In these patients, the best cut-off obtained for MAFLD-S was -1.5 (Se 89.5%, Sp 83.3%, NPV 94.6%, PPV 70.8%), 27.89 (Se 78.9%, Sp 78.6%, NPV 89.2%, PPV 62.5%) for FLI, 39.4 (Se 78.9%, Sp 64.2%, NPV 87.1%, PPV 49.9%) for HSI and -0.20 (Se 63.2%, Sp 88.1%, NPV 84.1%, PPV 70.6%) for CPN-CD.

## Discussion

To the best of our knowledge, this study is the first to specifically address the performance of several non-invasive steatosis scores in predicting MAFLD in patients with IBD. Our findings demonstrate that MAFLD-S, FLI, HSI, and CPN-CD can accurately predict MAFLD in a cohort of IBD patients, and therefore, be valuable tools in the management of these patients.

In 2020, Eslam et al. published an international expert consensus statement on a new definition of MAFLD to replace NAFLD nomenclature. This new term was considered more appropriate to reflect its pathogenesis and was deemed more helpful in patient stratification and management [3]. In their article, new positive diagnostic criteria for MAFLD were proposed, in which HS detected either by imaging techniques, blood biomarkers/scores (non-invasive steatosis tests) or by liver histology was the first step in the diagnostic flowchart, followed by the identification one of the following three clinical criteria: overweight/obesity, T2DM, or evidence of metabolic dysregulation [3].

MAFLD is a prevalent comorbidity among IBD patients [29]. For example, Arieira et al., reported a prevalence of HS ranging from 16.8% to 45.3% according to the diagnostic tool used (HSI, FLI or CAP by TE) [18]. Moreover, a recent meta-analysis revealed a pooled prevalence of 30.7% for NAFLD in patients with IBD globally, with a comparable risk observed in both CD and UC patients [5]. Similarly, in our study sample, the overall prevalence of MAFLD was 38.7%, and it did not significantly differ between CD and UC patients (43.0% vs. 31.1%, *P* = 0.141, respectively). Nonetheless, CD patients exhibited higher body weight and a more frequent occurrence of BMI ≥ 25 kg/m2 than UC patients, mirroring findings from a previous study [30]. This difference in weight status could also account for the elevated levels of triglycerides observed in CD patients in comparison to UC patients, as excess weight and obesity are associated with insulin resistance, triggering increased delivery of free fatty acids from adipose tissue to the liver [30].

The existing data regarding the mechanisms underlying the association between MAFLD and IBD are conflicting and not well understood [5, 7, 31]. In our sample, compared to non-MAFLD, MAFLD patients had a higher prevalence of HS and traditional metabolic risk factors, such as male sex, overweight/obesity (with higher weight, BMI and waist circumference), T2DM (with higher FPG), arterial hypertension, dyslipidemia (and higher triglycerides levels), underscoring the relevance of metabolic conditions. Moreover, we observed that patients who were older at the time of the study and at the onset of IBD diagnosis were more likely to have MAFLD. This aligns with the conclusions of several meta-analyses which suggested that advanced age and other metabolic risk factors might contribute to an elevated risk of NAFLD in IBD patients [5, 7, 31]. While a recent meta-analysis did not identify a history of IBD-related abdominal surgery as a significant risk factor for NAFLD in IBD, our results demonstrated that a greater proportion of MAFLD patients had undergone abdominal surgery compared to non-MAFLD patients [5, 7, 31]. This observation is consistent with data from earlier meta-analyses suggesting that IBD-related factors might also play a role in the development of MAFLD in IBD patients [5, 7, 31]. As our cohort had an average follow-up period of 9 years after surgery, it is plausible that these findings reflect post-surgery clinical improvement, potentially indicating enhanced nutritional status, increased appetite, and subsequent weight gain [32]. Identification of higher ALT and G-GT serum levels in MAFLD patients are in line with other studies. These elevated enzyme levels are believed to reflect hepatic expression of insulin resistance, which is commonly present in individuals with MAFLD. Importantly, even within the reference range, ALT and G-GT levels appear to correlate with the incidence of NAFLD and metabolic syndrome in a dose-dependent manner [33]. However, our study was not designed to draw robust conclusions about which mechanisms are more relevant in this context, so further research is needed to better understand the specific mechanisms linking MAFLD and IBD.

Early screening for MAFLD in the IBD population, ideally at the time of diagnosis, and particularly in patients with cardiovascular comorbidities, will not only enable the initiation of appropriate management for MAFLD and other associated comorbidities but also potentially impact the management of IBD itself. As far as we know, there is no robust data available to suggest that the management of MAFLD in IBD patients should differ from non-IBD patients. A stepwise approach, starting with dietary and lifestyle interventions, and if necessary, anti-obesity drugs or bariatric surgery should be considered to reduce liver and cardiovascular morbidity and mortality [34]. On the other hand, the presence of MAFLD may affect IBD treatment choices. Underlying liver steatosis is known to potentiate pathogenesis of drug-induced liver disease, and IBD patients with MAFLD who are treated with immunosuppressive agents are at a higher risk of developing liver injury as a second hit. Treatment strategies in these patients might include avoiding drugs associated with hepatic steatosis, such as methotrexate [35]. Additionally, overweight and obesity are currently the most frequent nutritional disorders in IBD patients and are common associated comorbidities in MAFLD patients. Obese IBD patients have been shown to experience more rapid clearance of immunomodulators and biological therapies, medical treatment failure, and negative surgical outcomes compared with non-obese patients. Nevertheless, there is currently a lack of data regarding whether interventions aimed at treating obesity can improve IBD outcomes [34].

In the last few decades, several NIT, including FLI and HSI have been developed and validated in certain populations for prediction of HS and NAFLD [11, 12, 17, 28]. FLI was originally created by Bedogni et al. as a simple score to predict HS in the general population, incorporating variables like waist circumference, BMI, triglycerides, and G-GT. A score of < 30 was associated with low risk, while ≥ 60 indicated a high risk of HS. [13] In the following years, many studies assessed its performance in predicting HS and NAFLD across different populations, comparing various HS diagnostic methods as the gold-standard. Although valuable for evaluating NAFLD in high-risk cohorts, FLI seems to possess limited capacity to definitively confirm or exclude NAFLD on an individual patient level [17]. HSI was created by Lee et al., based on AST, ALT, BMI, sex, and presence or absence of T2DM, aimed to predict NAFLD [14]. Since its inception, this score has been validated in diverse populations, encompassing individuals with human immunodeficiency virus (HIV) and those exhibiting insulin resistance, with comparisons made against alternative diagnostic methods [12]. Like FLI, available data implies that HSI serves as a practical tool for evaluating NAFLD in high-risk populations [28].

Following the redefinition of NAFLD to MAFLD, creation of new scores (such as MAFLD-S) or further validation of non-invasive tests used in NAFLD prediction was required for MAFLD prediction. Han AL et al. retrospectively evaluated performance of FLI and HSI for MAFLD prediction compared to computed tomography (CT)-diagnosed MAFLD, including 1300 adults aged ≥ 19 years who underwent CT scan from March 2012 to October 2019 in their institution [36]. FLI (AUROC, 0.793) and HSI (HSI 0.784) had acceptable performance in predicting MAFLD [36]. In other recent study using ultrasound as a reference standard, FLI (AUROC, 0.793) and HSI (AUROC 0.764) had good and similar performance [37]. Additionally, in a large cross-sectional survey in China including 135,436 patients, the FLI AUROC for MAFLD prediction obtained was 0.870 and 0.923 for men and women, respectively [27]. On the other hand, Ruiz-Manriquez et al., based on a cohort of 3357 adults from the general population of 5 Mexican states, developed the MAFLD-S score which includes the variables age, sex, BMI, T2DM, arterial hypertension and dyslipidemia [16]. They showed that this simple clinical tool could predict MAFLD with an AUROC of 0.852 ([95% CI, 0.828–0.877]) and with a sensitivity and a specificity of 78.8% and 82.8%, respectively, using an optimal cutoff. Even though it was created and internally validated in the original study, it was not externally validated. As far as we know, we have shown for the first time that MAFLD-S can accurately predict MAFLD in CD (AUROC, 0.949) and UC (AUROC, 0.877) patients, with high sensitivity (> 95% for CD and > 85% for UC) and high NPV (> 95% for CD and > 90% for UC) for the optimal cutoff (-1.6 for CD and -1.5 for UC). This outstanding performance in our IBD cohort should encourage other authors to further validate this score in other populations to clarify its usefulness in clinical practice.

Although many of the previous NIT were validated in high-risk groups, data regarding the performance of NIT in predicting NAFLD or MAFLD in IBD patients is limited. Bessissow et al. have addressed this issue. After applying HSI in 62 patients with IBD, obtained a AUROC of 0.74 (96% CI, 0.68– 0.80) for the prediction of HS using ultrasound as reference [38]. As far as we know, our group is the first to validated FLI (AUROC, 0.919 and AUROC, 0.797) and HSI (AUROC, 0.859 and AUROC, 0.713) in predicting MAFLD in CD and UC patients, respectively.

On the other hand, instead of using pre-existing scores, McHenry et al. created and internally validated CPNC-CD to predict NAFLD in CD patients. CPN-CD includes age, sex, ethnicity/race, ALT, BMI, known cardiometabolic diagnoses (arterial hypertension, T2DM, dyslipidemia), CD duration, and current use of azathioprine/6-mercaptopurine. They showed that CPN-CD had superior performance (AUROC, 0.85) compared to HSI (AUROC, 0.76) in predicting NAFLD in CD patients, using magnetic resonance proton density fat fraction as reference [15]. However, up until now, this score has not been externally validated, either for the new MAFLD criteria or in UC patients. In our cohort, we observed excellent discrimination in predicting MAFLD for both CD (AUROC, 0.818 [95% CI, 0.735–0.902]) and UC (AUROC, 0.818 [95% CI, 0.690–0.945]) patients. Interestingly, the remaining NIT evaluated (MAFLD-S, FLI and HSI) only incorporate well-established metabolic risk factors and mostly demonstrated higher or similar AUROC compared to CPNCD for the prediction of MAFLD in IBD patients, regardless of the type. Therefore, our results suggest that there is no need to use specific NIT for IBD patients that include the specificities of IBD.

Our study presents some limitations. This was a single center study, with a cross-sectional design with its possible inherent bias. Despite ultrasound is recommended by certain guidelines as the first-line tool for the diagnosis of HS in clinical practice, we used TE do detect HS using a validated CAP cut-off. This approach demonstrated high applicability (with only 4.2% unreliable/failure measurements), and is known to have comparable accuracy to ultrasound for detecting HS when liver biopsy is used as the reference standard [26]. Even though the prevalence of MAFLD was high in our sample, we were unable to measure all of the metabolic risk factors outlined in the MAFLD diagnosis criteria, namely the homeostasis model assessment of insulin resistance, 2-h post-load glucose levels, and HbA1c for T2DM diagnosis, which could potentially underestimate the prevalence of MAFLD. However, we included the most commonly used criteria in routine clinical practice, mitigating the potential impact of this hypothetical underestimation on our findings. Lastly, even though the criteria for diagnosing MAFLD no longer mandate the exclusion of other liver diseases, our study focused exclusively on IBD patients without coexisting liver diseases, limiting our ability to evaluate the performance of these scoring systems in the presence of other liver conditions.

Based on our findings, we suggest that non-invasive steatosis scores should be routinely employed in IBD patients, given their elevated pretest probability of MAFLD. In the daily busy setting of IBD clinics using these cheap, simple and not time-consuming scores could help in ruling out or in IBD patients that should be submitted to ultrasound or TE for assessment of HS. Our results indicate that MAFLD-S, FLI, HSI, and CPN-CD are effective tools for accurately predicting MAFLD in IBD patients. As limited evidence exists regarding the utility of non-invasive steatosis scores in IBD patients, our study represents a notable stride toward establishing the credibility of these diagnostic tools. Nevertheless, to more comprehensively elucidate their impact on clinical practice, large-scale, prospective, multicenter studies are needed applying these scores in IBD patients.

## Data Availability

All data generated or analyzed during this study are included in this published article.

## Abbreviations

ALT: alanine aminotransferase
AP: alkaline phosphatase
AST: aspartate aminotransferase
AUROC: area under the receiver-operating characteristic curve
BMI: body mass index
CAP: controlled attenuation parameter
CD: Crohn’s disease
CPN-CD: clinical prediction tool for nonalcoholic fatty liver disease in Crohn’s disease
CRP: c-reactive protein
FLI: fatty Liver Index
FPG: fasting plasma glucose
G-GT: γ-glutamyltransferase
HDL: high-density lipoprotein cholesterol
HS: hepatic steatosis
HSI: hepatic steatosis index
IBD: inflammatory bowel disease
IQR/M: interquartile range to median ratio
LDL: low-density cholesterol
LSM: liver stiffness measurement
MAFLD: metabolic dysfunction-associated fatty liver disease
MAFLD-S: metabolic dysfunction-associated fatty liver disease screening score
NAFLD: nonalcoholic fatty liver disease
NIT: non-invasive test
NPV: negative predictive value
PPV: positive predictive value
ROC: receiver-operating characteristic
Se: sensitivity
Sp: specificity
STROBE: Strengthening the Reporting of Observational Studies in Epidemiology
TC: total cholesterol
TE: transient elastography
T2DM: type 2 diabetes mellitus
